# Maternal High-Fat Diet Exposure During Gestation and Lactation Affects Intestinal Development in Suckling Rats

**DOI:** 10.3389/fphys.2021.693150

**Published:** 2021-07-08

**Authors:** Monika Słupecka-Ziemilska, Paulina Grzesiak, Paweł Kowalczyk, Piotr Wychowański, Jarosław Woliński

**Affiliations:** ^1^Department of Human Epigenetics, Mossakowski Medical Research Centre, Polish Academy of Sciences, Warsaw, Poland; ^2^Department of Animal Physiology, The Kielanowski Institute of Animal Physiology and Nutrition, Polish Academy of Sciences, Jabłonna, Poland; ^3^Department of Animal Nutrition, The Kielanowski Institute of Animal Physiology and Nutrition, Polish Academy of Sciences, Jabłonna, Poland; ^4^Department of Oral Surgery, Medical University of Warsaw, Warsaw, Poland

**Keywords:** intestine, development, programming, high fat diet, neonate, rat

## Abstract

Maternal health and diet influence metabolic status and play a crucial role in the development of metabolic function in offspring and their susceptibility to metabolic diseases in adulthood. The pathogenesis of various metabolic disorders is often associated with impairment in intestinal structure and function. Thus, the aim of the current study was to determine the effects of maternal exposure to a high fat diet (HFD), during gestation and lactation, on small intestinal growth and maturation in rat pups at 21 days old. Female, Wistar Han rats were fed either a breeding diet (BD) or high fat diet (HFD), from mating until the 21st day of lactation. Maternal HFD exposure increased body weight, BMI and adiposity. Compared to the maternal BD, HFD exposure influenced small intestine histomorphometry in a segment-dependent manner, changed the activity of brush border enzymes and had an impact on intestinal contractility *via* changes in cholinergic signaling. Moreover, offspring from the maternal HFD group had upregulated mRNA expression of cyclooxygenase (COX)-2, which plays a role in the inflammatory process. These results suggest that maternal HFD exposure, during gestation and lactation, programs the intestinal development of the offspring in a direction toward obesity as observed changes are also commonly reported in models of diet-induced obesity. The results also highlight the importance of maternal diet preferences in the process of developmental programming of metabolic diseases.

## Introduction

Development and growth during the early life period are influenced by maternal health and diet composition. Moreover, human epidemiological studies and animal experiments indicate that maternal health and diet influence metabolic status and play a crucial role in the development of metabolic function in offspring and their susceptibility to metabolic diseases in adulthood (Hanson and Gluckman, 2011). In particular, increased fat consumption, which is an attribute of the Western diet, is considered a major triggering factor of metabolic impairments such as obesity, type II diabetes, insulin resistance, dyslipidemia, and hypertension. The pathogenesis of obesity and metabolic syndrome is associated with impairments in intestinal absorption and motility (Miron and Dumitrascu, 2019). The intestine has been shown to be an important insulin-sensing tissue (Reynolds et al., 2015) and intestinal inflammation and oxidative stress may be key mechanisms involved in atherogenic dyslipidemia, which is turn triggers the development of metabolic syndrome and type 2 diabetes (Veilleux et al., 2014).

Surprisingly, very few studies have investigated the effects of maternal HFD exposure on neonatal gut development. A previous study in mice revealed changes in duodenal histology and alterations in the intestinal proteome of offspring from mothers fed a HFD during gestation and for 12 days postpartum (Suarez- Trujillo et al., 2019). It is important to note that the mechanism of maternal programming differs based on whether there is prenatal or postnatal exposure to changes in dietary composition. Maternal overnutrition and obesity during gestation is associated with morphological and functional alterations of the placenta, which in turn affects the transfer of glucose and amino acids from the dam into fetal circulation. Maternal fat intake has also been shown to influence the composition of the amniotic fluid, which is swallowed by the fetus or transported across the placenta (Friesen and Innis, 2006). Following birth, maternal obesity influences the composition of nutrients and bioactive components in the milk. For example, breast milk from overweight and obese mothers has higher total fat, glucose and insulin concentrations than milk from lean mothers (Ahuja et al., 2011). Moreover, the concentration of ghrelin and obestatin in breast milk changes following maternal exposure to a HFD during gestation and lactation and maternal body mass index (BMI) has been shown to be associated with the level of adipokines in breast milk (Savino et al., 2009; Newburg et al., 2010). It should be also highlighted that changes in maternal nutrition during pregnancy and lactation may have a substantial impact on offspring physiology and susceptibility to disease by influence on microbiota abundance and function (Chu et al., 2016). Both prenatal and postnatal conditions are crucial for the development of the gastrointestinal tract of rodents, which starts during gestation and is not fully mature until weaning and thus still sensitive to variations in the content of nutrients and biologically active substances in mothers’ milk. We hypothesized that early life exposure to a maternal HFD affects the development of the gastrointestinal tract in offspring, thus impacting its function.

Our objective was to determine the effects of exposure to a maternal HFD, during gestation and lactation, on offspring small intestinal growth and maturation at weaning, in order to better understand how the maternal diet shapes intestinal function and may impact on the long-term health of offspring.

## Materials and Methods

### Animals

The experiments and treatments were conducted in compliance with European Union regulations concerning the protection of experimental animals. The 3rd Local Animal Ethics Committee located in Warsaw approved the study protocol. For the purpose of the study 14 male and 14 female Wistar Han rats (13 weeks old) were purchased from the Center of Experimental Medicine at the Medical University of Bialystok. After two weeks of acclimatization, the females were examined with a vaginal impedance checker (Muromachi Kikai Co., Ltd.) for the precise determination of the stage of estrous for mating time. Based upon these measurements, female rats at the appropriate point in the estrous cycle were selected for breeding. On mating day, the rats were also randomly allocated to either a high-fat diet (HFD, 30% fat; 4.7 kcal/g; *n* = 7) or standard breeding diet group (BD; 5% fat; 3.1 kcal/g; *n* = 7) [diets were purchased from Wytwórnia Pasz Morawski (Poland)]. Main components of diets given to rats are presented in Table 1. Porcine lard was used as the source of fat in the HFD. The next day the mated females were examined for the presence of a vaginal plug. Females with a vaginal plug (*n* = 12, 6 for each diet) were further selected for the experiment and this was considered as day 1 of gestation. On day 14 of gestation, the females were separated from the males. The day upon which females gave birth was considered as day 1 of lactation. All females gave birth 21 ± 1 days after mating. On the first day of lactation, the litters (*n* = 6 for each maternal diet) were standardized to ten pups. For the purposes of a parallel study, on day 14 of lactation, two pups from each litter were sampled for blood and then sacrificed (*n* = 12 for each maternal diet). The rest of the pups stayed with their dams and continued to be used in the experiment until the 21st day of lactation. Body weight, blood glucose and plasma triglycerides of rat dams and their offspring till the 21st day of lactation was previously published (Słupecka et al., 2016). Maternal diet had no effect on the litter size (data not shown).

**TABLE 1:** Composition of diets fed to rats.

Amount per kg	Standard breeding diet (BD, 5% fat)	High-fat diet (HFD, 30% fat)
Dry matter	885 g	918 g
Crude protein	222 g	190 g
Crude lipids	50 g	297 g
Carbohydrates	572 g	401 g
Crude fiber	46 g	32 g
Crude ash	41 g	30 g
Metabolic energy	12,8 MJ/3057 kcal	19 MJ/4658 kcal

On the 21st day of lactation, all rat pups were weaned and one male and one female rat pup were randomly selected from each of 12 litters (*n* = 12 animals for each maternal diet, 6 females, and 6 males), weighed and subjected to a DXA scan under isoflurane inhalation (Norland XR-800TM densitometer scanner with a “Research Scan” type, Norland, A Cooper Surgical Company, Fort Atkinson, WI, United States). The scans were obtained using standard procedures supplied by the manufacturer for scanning and analysis. Following the DXA scan, the rats were sacrificed using an overdose of CO_2_.

The duodenum (approximately 2 cm from the pylorus ring, on the anal side), middle jejunum (approximately halfway between the anal side of the duodenojejunal flexure and the oral side of the cecum) and ileum (approximately 2 cm on the oral side of the cecum) were transected, and samples of each section were collected and immediately fixed in a 10% neutral formalin solution. The middle part of the jejunum was deep-frozen in liquid nitrogen (–80°C) for further analyses: brush border enzyme activity and analysis of gene expression. Additionally, 15 mm long duodenal and middle jejunum segments were placed in cold Krebs–Henseleit buffer (KCl 4.7, KH2PO4 1.2, MgSO4 1.2, CaCl2 1.25, NaHCO3 25, glucose) for further organ bath studies.

### Intestinal Contractility *in vitro*

The whole wall intestinal segments placed in cold Krebs–Henseleit buffer were then fixed vertically in 25 ml organ bath chambers (Letica Scientific Instruments, Spain), filled with Krebs–Henseleit solution (37°C, pH 7.4) and continuously saturated with carbogen (95% O2, 5% CO_2_). The intestinal segments were attached to isometric transducers (Letica Scientific Instruments, Spain) under a load of 0.5 g. The transducers were coupled with a PowerLab recording system (ADInstruments, Sydney, Australia). The tissues were allowed to equilibrate for 30 min (the solution in the chambers was changed once after 15 min) to regain spontaneous activity. The segments were then subjected to a procedure which started with the addition of ACh 10^–5^ M to assess the viability of the preparations. ACh was left in the solution for 1 min, after which the tissues were washed and allowed to equilibrate. Next, ACh-stimulated contractility was recorded. ACh-stimulated contractility was recorded as the response to ACh 10^–5^ M. Additionally, in some experiments, before ACh was added, jejunal strips were pre-treated with atropine (ATR, 10^–5^ M) or with tetradotoxin (TTX, 10^–5^ M). Each experiment was completed by the administration of ACh 10^–5^ M in order to check the viability of the tissue, followed by isoproterenol (10^–5^ M) in order to control its relaxation.

### Functional Development of Small Intestinal Mucosa

A segment of the middle jejunum was examined for brush border enzyme activity. After thawing, samples of the intestinal mucosa were homogenized in cold distilled water (1 g intestinal mucosa/5 ml distilled water) and centrifuged for 5 min at 1,000 *g* at 4°C. The protein content was then determined as described by Hartree (1972), with BSA as the standard. The activities of aminopeptidase A and N were assayed with N̈-glutamyl- *p*-nitroanilide and N̈-leucyl-*p*-nitroanilide as substrates, respectively (Maroux et al., 1973) and that of dipeptidyl peptidase IV was assayed with glycyl-N̈-prolyl-*p*-nitroanilide (Nagatsu et al., 1976). The resulting enzymatic units (IU) are expressed as μmol *p*-nitroanilide released/min at 37°C. Lactase, maltase and sucrase activities were determined as previously described (Dahlqvist, 1984), with minor modifications.

### Small Intestine Mucosa Remodeling

Preparation of the intestinal samples for histomorphometry and immunofluorescence staining of the intestinal wall was performed on paraffin embedded sections, as previously described in detail by Słupecka et al. (2012). Briefly, for intestinal histomorphometry studies, slides were stained with hematoxylin and eosin. Villi length, crypt depth and, thickness of mucosal layer (from the tip of the villi to the bottom of the crypt) were visualized using a light microscope (Axioskop 40, Zeiss, Germany) and analyzed using Axio Vision 4.2 Release software (Zeiss, Germany).

The number of apoptotic epithelial cells was determined using a TUNEL assay (terminal deoxynucleotidyl transferase-mediated dUTP nick end labeling). The procedure was performed according to the manufacturer’s protocol for the assay (ApopTag^®^ Red in Sity Apoptosis Detection Kit, Merck Millipore, Darmstadt, Germany United States). Slides were visualized using a microscope under 400x magnification (LSM 5 Pascal, Zeiss, Germany). The apoptotic index was estimated separately for crypt and villi area. The apoptotic index was expressed as the number of apoptotic cells on all epithelial cells of villi and crypt, respectively. Proliferating crypt cells were immunostained for rabbit polyclonal anti-Ki67 antibodies (Abcam, United Kingdom), 50 times diluted in 1% BSA-PBS and processed according to the manufacturer’s protocol for EnVision + system (DakoCytomation, Denmark), providing secondary antibodies conjugated with HRP enzyme and the chromogen (diaminobenzidine) as the substrate for the HRP enzyme. The slides were counterstained with hematoxylin. The mitotic index was calculated as the number of Ki67 positive cells on all epithelial cells of the crypt cross-section. For all abovementioned analysis a minimum of 15 well-oriented and intact villi and crypts were randomly selected per slide by an investigator blinded to treatment allocation.

### Cox-2 Mediated Inflammation

To examine the role of cyclooxygenase (Cox)-2-mediated inflammation in the development of the rar intestine, the gene expression of Cox-2 was measured using Real Time RT-PCR. Briefly, total RNA was extracted from tissue from the middle part of the jejunum of each rat (frozen in liquid nitrogen immediately after euthanasia) using the PureLink RNA kit (Invitrogen, United States). RNA quantity and purity were determined using a NanoDrop microspectrophotometer ND-1000 (Wilmington, DE). One μg of total RNA was reverse-transcribed to high capacity cDNA using a reverse transcription kit, including a mixture of random primers (Applied Biosystems, Foster City, CA). Real time quantitative RT-PCR analysis was carried out by SYBR Green I dye detection (Applied Biosystems, Foster City, CA), using the real time detection system (Bio-Rad).

Real-time PCR primers designed with PrimerExpress software were as follows:

• Cox-2 F, GATTGACAGCCCACCAACTT Exon 4, NM_ 017232
• Cox-2 R, CGGGATGAACTCTCTCCTCA Exon 5

Primers were synthesized in a DNA Sequencing and Oligonucleotide Synthesis Laboratory IBB, PAS (Poland). The following optimizing conditions were applied: 1 min at 48°C, 7 min, and 30 s at 95°C and 45 cycles of 20 s at 95°C and 35 s at 62°C in which an optical acquirement were performed. Melt curves were performed upon completion of the cycles to ensure absence of non-specific products. Quantification was performed by normalizing cycle threshold (*Ct*) values with the Cox2 control gene 18 s, and analysis was carried out using the 2−ΔΔ*CT* method (Livak and Schmittgen, 2001).

### Data Analysis

The obtained data were analyzed using unpaired *t*-test or Mann-Whitney test (*p* < 0.05), (Prism 6 for Mac OS X, Version 6.0h, GraphPad Software, Inc., United States).

## Results

### Sex-Specific Differences

Both sexes were independently assessed. However, analyses of studied parameters taking into account sex variation did not identify any sex-specific differences. In consequence, to simplify, the data presentation results from both sexes were combined and presented as offspring data.

### Body Weight, Adiposity and Small Intestine Morphometry

**FIGURE 1:**
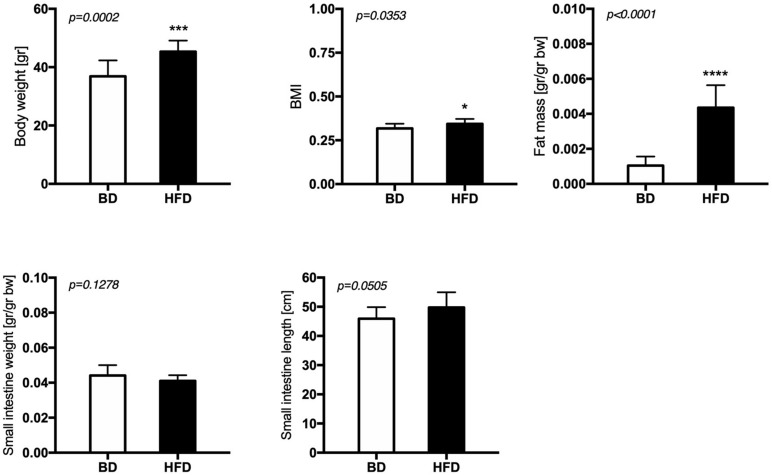
The effect of maternal HFD exposure during gestation and lactation on the body weight, adiposity and intestinal morphology. BD- 21-day old rats from dams fed with breeding diet during gestation and lactation (*n* = 12); HFD- 21-day old rats from dams fed with high fat diet during gestation and lactation (*n* = 12); values are given as means ± SEM; unpaired *t*-test or Mann-Whitney test was used to show statistical differences between the study groups * indicate statistical difference between the groups.

Maternal exposure to a HFD during gestation and lactation increased the body weight (*p* = 0.0002), BMI (*p* = 0.0353), and fat mass (*p* < 0.0001), but had no effect on small intestine weight and length of offspring from HFD mothers, compared to offspring from mothers fed the breeding diet (BD) (Figure 1).

### Small Intestinal Contractility *in vitro*

**FIGURE 2:**
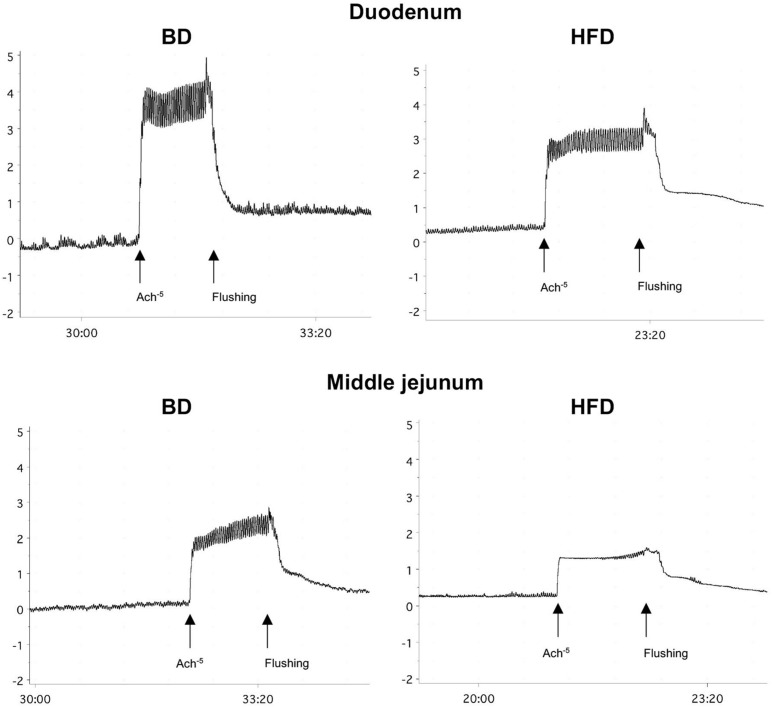
Representative tracing of ACh-evoked contraction in duodenal and middle jejunum segments. BD- 21-day old rats from dams fed with breeding diet during gestation and lactation; HFD- 21-day old rats from dams fed with high fat diet during gestation and lactation.

Maternal HFD exposure during gestation and lactation resulted in a significantly lower amplitude of contraction in the duodenum and middle jejunum after treatment with ACh (*p* = 0.0668, *p* = 0.0081, respectively), but had no effect on the contraction of segments treated with TTX and ATR (*p* > 0.05). In contrast, the frequency of contraction was significantly increased in both intestinal segments and for all mediators (ACh, TTX, ATR) in the HFD offspring group (*p* < 0.05), compared to the BD offspring (Figure 2 and Table 2).

**TABLE 2:** The effect of acetylcholine (Ach^–5^ M), tetradotoxin (TTX^–6^ M), and atropine (ATR^–6^ M) on the amplitude (mm) **(A)**, and frequency (number of contractions per 1 s) **(B)** of spontaneous contraction according to the small intestinal segment examined and treatments.

	Duodenum				Middle jejunum			
	Basal	ACh^–5^	TTX^–6^	ATR^–6^	Basal	ACh^–5^	TTX^–6^	ATR^–6^
**(A)**								
BD	0.64 ± 0.16	5.28 ± 0.92	0.40 ± 0.22	0.33 ± 0.13	0.51 ± 0.18	4.50 ± 1.37	0.15 ± 0.09	0.30 ± 0.18
HFD	0.62 ± 0.22	3.41 ± 1.38	0.52 ± 0.30	0.64 ± 0.34	0.10 ± 0.05**	1.39 ± 0.83**	0.12 ± 0.06	0.15 ± 0.05
*P*	0.8407	0.0668	0.4221	0.1833	0.0095	0.0081	0.5916	0.2364
**(B)**								
BD	0.34 ± 0.05	0.86 ± 0.06	1.34 ± 0.04	2.34 ± 0.15	0.42 ± 0.07	1.07 ± 0.08	3.04 ± 0.54	3.29 ± 0.74
HFD	0.41 ± 0.06	7.80 ± 0.86****	6.77 ± 2.14**	4.47 ± 1.15*	0.48 ± 0.10	5.83 ± 0.84***	5.83 ± 0.84*	6.03 ± 0.67*
*P*	0.8235	<0.0001	0.0023	0.0117	0.8617	0.0002	0.0182	0.0158

BD- 21-day old rats from dams fed with breeding diet during gestatation and lactation (n = 12); HFD- 21-day old rats from dams fed with high fat diet during gestation and lactation (n = 12); different superscript letters indicate statistical difference between the groups within type of contraction; *indicates statistical differences between basal ACh, TTX or ATR- blocked contraction; unpaired t-test or Mann-Whitney test was used to show statistical differences between the study groups (*P ≤ 0.05, **P ≤ 0.01, ***P ≤ 0.001, ****P ≤ 0.0001).

### Functional Development of the Small Intestine Mucosa

**FIGURE 3:**
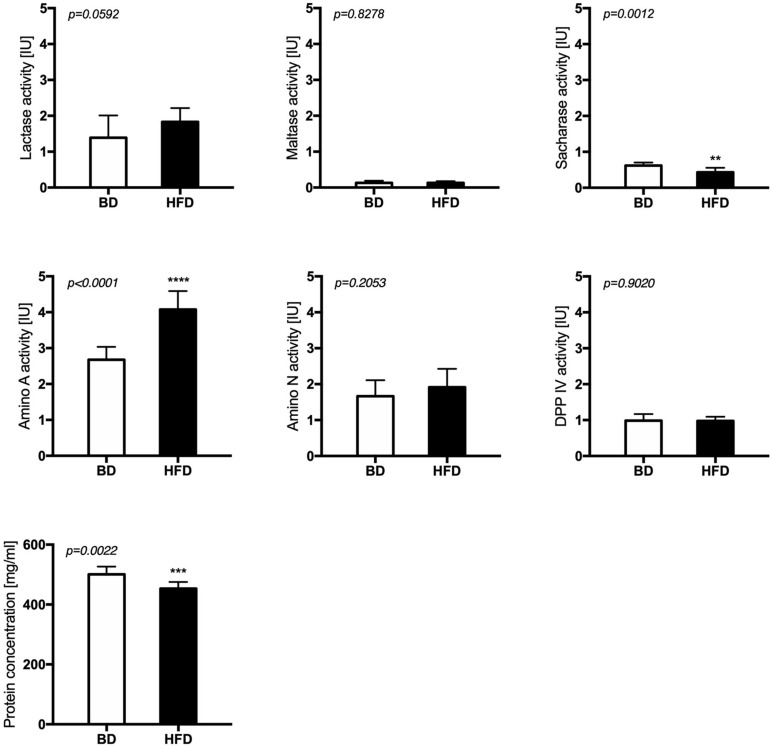
The activity of brush border enzymes after maternal HFD exposure during gestation and lactation. BD- 21-day old rats from dams fed with breeding diet during gestation and lactation (*n* = 12); HFD- 21-day old rats from dams fed with high fat diet during gestation and lactation (*n* = 12); values are given as means ± SEM; unpaired *t*-test or Mann-Whitney test was used to show statistical differences between the study groups * indicate statistical difference between the groups.

Maternal HFD exposure during gestation and lactation significantly decreased the activities of sacharase (*p* = 0.0012) and aminopeptidase A (*p* < 0.0001), as well as the total protein content (*p* = 0.0022) in the mucosal samples from the middle jejunum of HFD offspring, compared to offspring from mothers fed a breeding diet (BD). A trend for increased lactase activity (*p* = 0.0592) was also observed in HFD offspring group (Figure 3).

### Small Intestine Mucosa Remodeling

**FIGURE 4:**
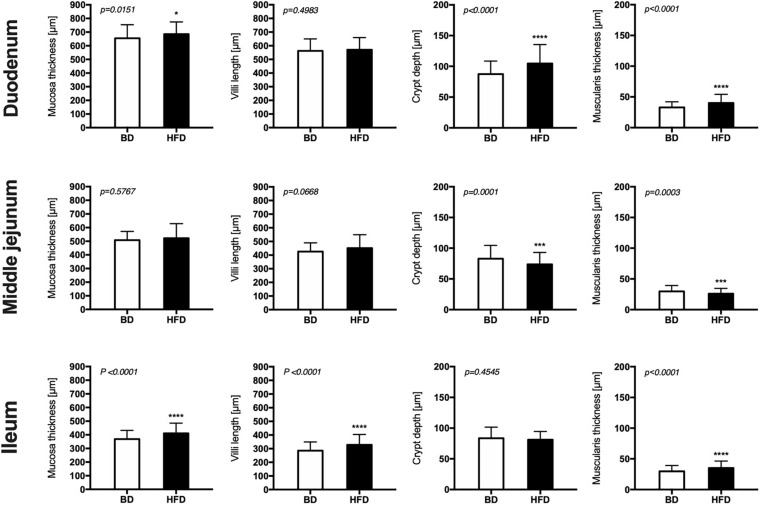
The effect of maternal HFD exposure during gestation and lactation on intestinal histomorphometry in suckling rats. BD- 21-day old rats from dams fed with breeding diet during gestation and lactation (*n* = 12); HFD- 21-day old rats from dams fed with high fat diet during gestation and lactation (*n* = 12); values are given as means ± SEM; unpaired *t*-test or Mann-Whitney test was used to show statistical differences between the study groups * indicate statistical difference between the groups.

**FIGURE 5:**
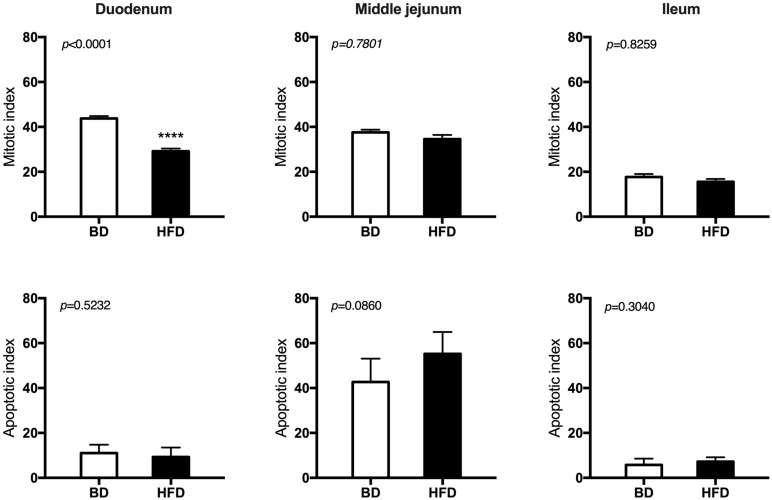
The effect of maternal HFD exposure during gestation and lactation on intestinal epithelium renewal in suckling rats. BD- 21-day old rats from dams fed with breeding diet during gestation and lactation (*n* = 12); HFD- 21-day old rats from dams fed with high fat diet during gestation and lactation (*n* = 12); values are given as means ± SEM; unpaired *t*-test or Mann-Whitney test was used to show statistical differences between the study groups * indicate statistical difference between the groups.

**FIGURE 6:**
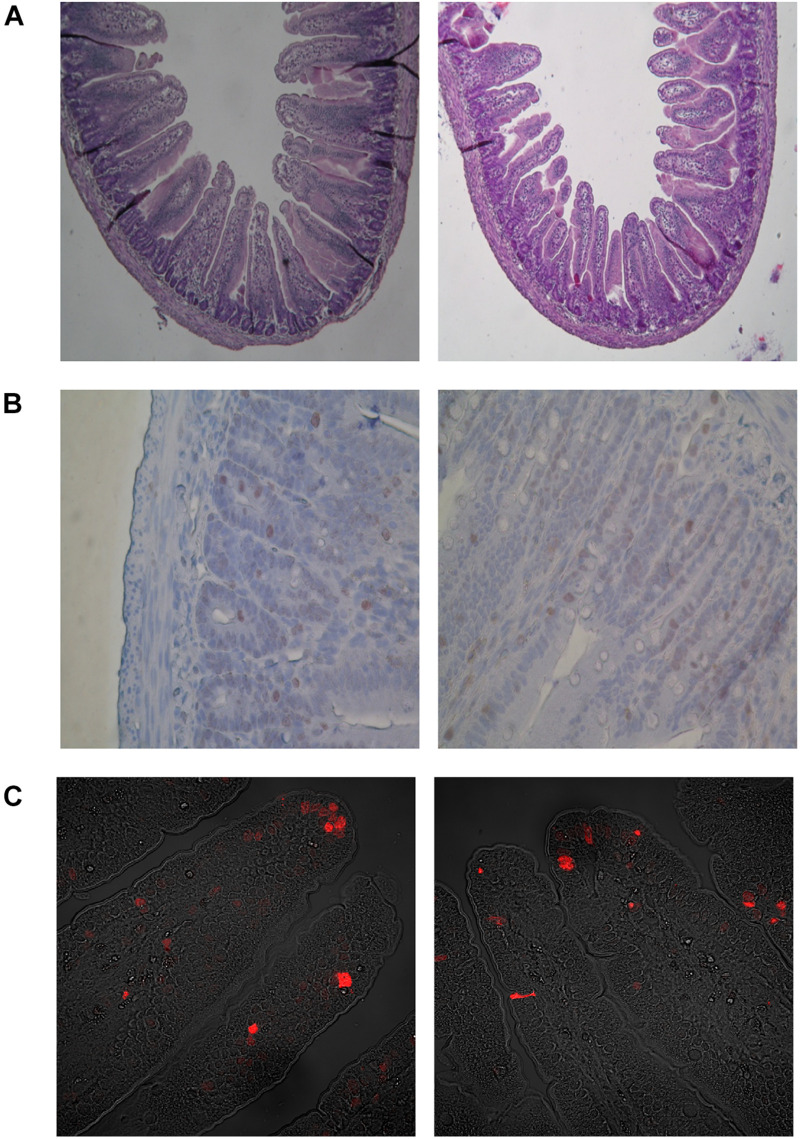
Microscopic images in representative cross-sections from the middle small intestine mucosa of suckling rats. **(A)** Histometry of small intestinal wall in suckling rats (light microscope, 100x). **(B)** Mitosis of crypt small intestinal epithelial cells (light microscope, 400x). **(C)** Apoptosis of villous small intestinal epithelial cells (confocal microscope, 400x). BD- 21-day-old rats from dams fed with breeding diet during gestation and lactation; HFD- 21-day-old rats from dams fed with high fat diet during gestation and lactation.

Histomorphometric analysis of the intestinal wall revealed a significant increase in the thickness of the mucosa layer and in the depth of the crypts, as well as a decrease in the thickness of muscularis layer in the duodenum of HFD offspring compared to BD offspring (Figure 4). Additionally, a decreased proliferation rate was also observed. In the middle jejunum, maternal HFD exposure decreased crypt depth and the thickness of muscularis layer in HFD offspring, with no changes in the rate of proliferation and apoptosis. In the ileum, a significant increase in the thickness of both the mucosa and the muscularis layer, as well as longer villi were observed in the HFD offspring, compared to the BD offspring. No changes in proliferation and apoptosis rate were observed in the ileum of both groups of rats (Figures 5, 6).

### Cox-2 Gene Expression

**FIGURE 7:**
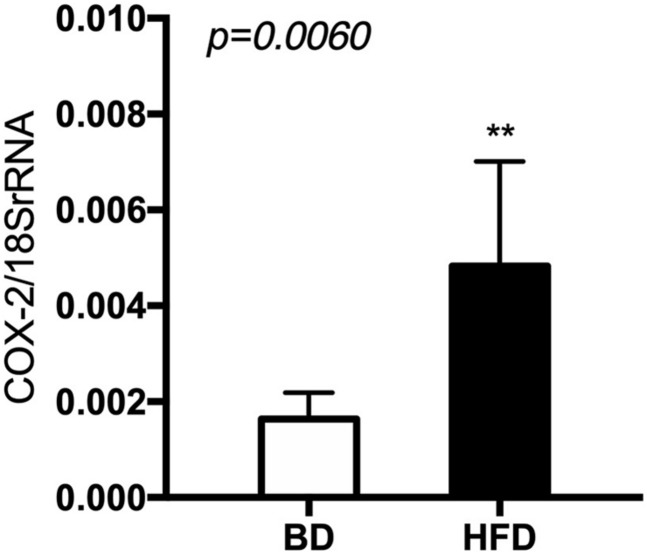
The level of Cox-2 mRNA in relation to 18 S rRNA in extracts from rat’s small intestine. BD- 21-day old rats from dams fed with breeding diet during gestation and lactation (*n* = 12); HFD- 21-day old rats from dams fed with high fat diet during gestation and lactation (*n* = 12); values are given as means ± SEM; unpaired *t*-test or Mann-Whitney test was used to show statistical differences between the study groups * indicate statistical difference between the groups.

The expression of cyclooxygenase (Cox)-2 mRNA was significantly increased (*p* < 0.006) in the HFD, compared to the BD offspring (Figure 7).

## Discussion

In order to better understand the programming of metabolic disorders and obesity development, we designed the current study in which female, Wistar Han rats were exposed to a high fat diet (HFD) during gestation until the end of lactation, that is until weaning on the 21st day of life of the offspring.

Our results demonstrated that exposure to a HFD *in utero* and during the suckling period increases offspring body weight and adiposity, as well as influencing small intestine structure and function. In particular, exposure to maternal HFD influences intestinal histomorphometry in a segment-dependent manner, driven by the local niche environment specific o each intestinal segment (Zhou et al., 2018). Over and above the changes in intestinal histomorphometry, changes in the balance between proliferation and apoptosis were also observed in the duodenum. The observed hypertrophy of the duodenal and ileal mucosa of HFD rat offspring in the current study may be, at least to some extent, a result of changes in intestinal digestion and absorption, manifested by the increased activity of aminopeptidase A and decreased activity of sucrase. These results are in agreement with those of a previous study on the role of maternal HFD exposure during gestation and for 12 days postpartum in mice, which revealed significant changes in duodenal histomorphometry and the proteome of the mice. Exposure to maternal HFD during gestation and lactation increased the expression of proteins involved in fat digestion, absorption and fatty acid transport and decreased the level of proteins that regulate fatty acid biosynthesis in the rat pups’ duodenum. Increased villi height was observed in the HFD offspring, with no effect on proliferation, which was explained by the increased extracellular matrix protein deposition as a consistent response to the long-term exposure to a HFD, accompanied by obesity development in other tissues (Suarez- Trujillo et al., 2019). Another study involving the administration of a high fat diet to mice also observed hypertrophy of the intestinal mucosa, with no differences in the intensity of proliferative activity of crypt stem cells (Zhou et al., 2018). The authors suggested that this increase in epithelial mass was reflected by an increase in the number of intestinal epithelial cells (IESCs) and goblet cells (Zhou et al., 2018). In the current study, the number of IESCs and goblet cells was not measured, but we did analyze intestinal contractility, which revealed a significant decrease in the amplitude of contraction in the HFD offspring after treatment with ACh, which in turn may result in prolonged availability of nutrients in the intestinal lumen and thus the possibility of increased growth of intestinal epithelial cells. It should be mentioned that slower motor activity within the intestine, may also lead to food stagnation in the small intestine, causing small intestinal bacterial overgrowth (SIBO), another factor leading to the development of metabolic disorders and obesity. Decreased spontaneous amplitude of contraction of an isolated segment of the duodenum has previously been reported, following exposure to a high fat diet (Fu et al., 2014). Our results showed that early life exposure to a maternal HFD had no effect on spontaneous contraction of the intestine, but significantly influenced the cholinergic system in the intestine. Whether the results obtained are related to the number of cholinergic receptors located in the gut or the receptor sensitivity requires further investigation. Previous studies have confirmed the anti-inflammatory effect of ACh (Goverse et al., 2016) and that cholinergic signaling and the expression of ACh- receptors in the enteric nervous system (ENS) have a putative role in gut inflammation (Lakhan and Kirchgessner, 2011), as well as diet-induced obesity (Fu et al., 2014). Based on our results, we speculated that changes in the ACh responsiveness in the HFD offspring may be a programmed effect of the maternal HFD exposure, which in turn may be involved in the development of intestinal immune-mediated diseases. Furthermore, a previous study on rat offspring exposure to HFD reported an increased level of haptoglobin, an acute-phase protein, and significant increase in proinflammatory cytokine expression in rat offspring of HF-fed rat dams (Fåk et al., 2012; Reynolds et al., 2015).

The current study also investigated the effect of early life exposure to HFD on intestinal inflammation, through the measurement of the gene expression of Cox-2, an important mediator of inflammation and cell differentiation. Previous studies support the concept that HFD-induced inflammatory changes within the small intestine contribute to the development of or susceptibility to insulin resistance and obesity (Ding and Lund, 2011). Cox-2 is involved in maintaining the mucosal integrity of the small intestine (Ohno et al., 2004) and Cox-2 mediated inflammation may contribute to the development of insulin-resistance and type 2 diabetes mellitus in some individuals (Konheim and Wolford, 2003; Veilleux et al., 2014). Enhanced mRNA expression of Cox-2 has been observed in the epididymal fat of HFD-induced obese rats (Hsieh et al., 2009). We observed a significant increase in Cox-2 expression in the small intestine of the HFD rat offspring in the current study, which may be a precursor for the occurrence of metabolic diseases later on. Although some previous studies claim that Cox-2 upregulates through intestinal hypermotility (Ohno et al., 2004), which is in contradiction with the results of the present study from the organ bath studies, other studies suggest a more complex mechanism where inflammation at one site of the gut may possibly alter gut motility at another, non-inflamed site (Dimidi et al., 2017).

In summary, our results indicate substantial changes in the small intestine structure and function, which are typical following HFD feeding, including hypertrophy of the small intestine mucosa without an increase in proliferation rate, as well as increased expression of the inflammatory mediator Cox-2, decreased activity of sucrase, and changes in cholinergic signaling in the gut, which in turn influenced gut motility. Taken together, these changes may facilitate the development of metabolic disorders including obesity later in adult life.

## Data Availability Statement

The datasets presented in this study can be found in online repositories. The names of the repository/repositories and accession number(s) can be found below: https://www.ncbi.nlm.nih.gov/genbank/, NM_017232.

## Ethics Statement

The animal study was reviewed and approved by the 3rd Local Animal Ethics Committee located in Warsaw.

## Author Contributions

MS-Z designed the study and wrote the manuscript. MS-Z, PG, JW, PK, and PW were involved in carrying out the study. JW and MS-Z conducted data analyses for the study. All authors contributed to manuscript revision and read and approved the final version of manuscript.

## Conflict of Interest

The authors declare that the research was conducted in the absence of any commercial or financial relationships that could be construed as a potential conflict of interest.
